# Resuscitation of Newborn Piglets. Short-Term Influence of FiO_2_ on Matrix Metalloproteinases, Caspase-3 and BDNF

**DOI:** 10.1371/journal.pone.0014261

**Published:** 2010-12-09

**Authors:** Rønnaug Solberg, Else Marit Løberg, Jannicke H. Andresen, Marianne S. Wright, Eliane Charrat, Michel Khrestchatisky, Santiago Rivera, Ola Didrik Saugstad

**Affiliations:** 1 Department of Paediatric Research, University of Oslo and Oslo University Hospital, Rikshospitalet, Oslo, Norway; 2 Department for Surgical Research, University of Oslo and Oslo University Hospital, Rikshospitalet, Oslo, Norway; 3 Department of Pediatrics, Vestfold Central Hospital, Tønsberg, Norway; 4 Department of Pathology, Oslo University Hospital, Ullevål, Oslo, Norway; 5 UMR 6184 NICN, CNRS-Université d'Aix-Marseille II Faculté de Médicine, Marseille, France; University of Giessen Lung Center, Germany

## Abstract

**Background:**

Perinatal hypoxia-ischemia is a major cause of mortality and cerebral morbidity, and using oxygen during newborn resuscitation may further harm the brain. The aim was to examine how supplementary oxygen used for newborn resuscitation would influence early brain tissue injury, cell death and repair processes and the regulation of genes related to apoptosis, neurodegeneration and neuroprotection.

**Methods and Findings:**

Anesthetized newborn piglets were subjected to global hypoxia and then randomly assigned to resuscitation with 21%, 40% or 100% O_2_ for 30 min and followed for 9 h. An additional group received 100% O_2_ for 30 min without preceding hypoxia. The left hemisphere was used for histopathology and immunohistochemistry and the right hemisphere was used for *in situ* zymography in the corpus striatum; gene expression and the activity of various relevant biofactors were measured in the frontal cortex. There was an increase in the net matrix metalloproteinase gelatinolytic activity in the corpus striatum from piglets resuscitated with 100% oxygen vs. 21%. Hematoxylin-eosin (HE) staining revealed no significant changes. Nine hours after oxygen-assisted resuscitation, caspase-3 expression and activity was increased by 30–40% in the 100% O_2_ group (n = 9/10) vs. the 21% O_2_ group (n = 10; p<0.04), whereas brain-derived neurotrophic factor (BDNF) activity was decreased by 65% p<0.03.

**Conclusions:**

The use of 100% oxygen for resuscitation resulted in increased potentially harmful proteolytic activities and attenuated BDNF activity when compared with 21%. Although there were no significant changes in short term cell loss, hyperoxia seems to cause an early imbalance between neuroprotective and neurotoxic mechanisms that might compromise the final pathological outcome.

## Introduction

Perinatal hypoxic-ischemic (HI) brain damage is a major cause of neuronal and behavioral deficits [1]. HI is an injurious event that may precipitate a cascade of biochemical processes, which can lead to neuronal cell death after hours or days [2]. The aim of resuscitation is to prevent death and adverse long-term neurodevelopmental impairment. Recent research has shown that using extra oxygen for newborn resuscitation negatively influences both morbidity and mortality [3]–[8]. Hyperoxia causes apoptotic cell death in the developing brain and there is a time window within which various neuronal populations are more vulnerable to hyperoxia-induced cell death [9]. The homeostasis of the central nervous system (CNS) is strictly regulated by the blood-brain barrier (BBB) and the blood-cerebrospinal fluid barriers. Matrix metalloproteinases (MMPs) play a significant role in brain damage and repair after hypoxia-reoxygenation because they mediate the disruption of the BBB, resulting in neurovascular dysfunction and vasogenic edema. MMPs also regulate tissue inflammation in response to oxidative stress [10], [11]; MMP-9 has been shown to induce neuronal death [12]–[14], and MMP-2 has also been found to play a role in neuronal damage [15], [16]. However, in the delayed phases after injury, MMPs and other proteases may also play beneficial roles by modulating the extracellular matrix (ECM) and trophic factors in the brain parenchyma and at the neurovascular interface [11], [17]. One of the neurotrophins, brain-derived neurotrophic factor (BDNF), plays a crucial role in neuronal survival and maintenance, neurogenesis, learning and memory [18]–[21]. In humans, BDNF mRNA levels were found to be highest in neonates and to decrease with age [18]. Within the neonatal period there is also a change in BDNF such that normal term newborns experience a specific BDNF increase in serum levels from birth to day four, possibly reflecting neuroprotection against perinatal stress and hypoxia [22]. Such neuroprotective action may be mediated by the blockade of caspase-3 by BDNF [19], [23]. Indeed, this intracellular protease, which plays a major role in cell death, is strongly up regulated in the immature brain [24] and activated after hypoxia in the cerebral cortex [25].

The aim of this study was to examine the possible detrimental effects on the developing brain at the onset of the secondary energy failure after hypoxia and oxygen-assisted neonatal resuscitation. The newborn piglet provides a naturalistic model for the study of perinatal asphyxia. Before three days of age, the piglet's CNS maturation is similar to that of term newborn infants [26] and displays an inter-individual genetic diversity comparable to that of newborn humans. With an observation time of 9 h, we sought to detect early histopathological changes, a possible rise in the net MMP activity and more persistent gene changes in the expression of genes related to apoptosis, neurodegeneration or neuroprotection. Although the primary focus was to study the differences between using 21%, 40% or 100% oxygen for resuscitation, we also wanted to study how a brief exposure to hyperoxia without preceding hypoxia would influence gene regulation.

## Results

### Background data

There were no significant differences across groups 1, -2 and - 3 with respect to hemoglobin, body weight, age, gender, and time of hypoxia.

pH, base excess (BE), mean arterial blood pressure (MABP), pCO_2_ and heart rate (HR) were also not significantly different between the comparable groups (Tab.1). Most of the background data in Table 1 have been reported in another publication from our group [27], but there are no overlaps in the results.

**Table 1:** Background data.

	Control	Hyperoxia	21%	40%	100%
Weight (g)	1858(130)	1870 (143)	1873 (108)	1842 (108)	1852 (117)
Age (h)	35.5 (1)	35.1 (2.8)	35.1 (2.1)	32.8 (4)	32.8 (5.8)
Gender M/F	3/3	5/6	5/5	6/6	5/5
Hb g/100 mL start	7.2 (0.8)	7.3 (1.5)	7.1 (0.9)	6.9 (1.3)	6.9 (1)
End	6.5 (1.5)	6.2 (1.5)	6.4 (1.4)	6.1 (1.3)	6.1 (1.0)
Hypoxia (min)	0	0	33.7 (8.4)	37.8 (15.3)	42.3 (15.2)
**pH** start	7.44(0.05)	7.40 (0.05)	7.41 (0.06)	7.43 (0.05)	7.44 (0.06)
end hypoxia	*7.42 (0.03)*	*7.39 (0.2)*	6.91 (0.09)	6.91 (0.1)	6.94 (0.9)
end resuscitation	*7.43 (0.04)*	*7.46 (0.06)*	7.17 (0.09)	7.18 (0.06)	7.25 (0.11)
2h>resuscitation	7.45(0.05)	7.42 (0.08)	7.37 (0.05)	7.37 (0.05)	7.40 (0.08)
5h>	7.45(0.06)	7.38 (0.09)	7.35 (0.06)	7.37 (0.06)	7.43 (0.07)
9h>	7.42(0.08)	7.39 (0.11)	7.33 (0.06)	7.36 (0.08)	7.38 (0.11)
**BE** mmol/L start	1.1 (2.9)	1.5 (3.2)	−0.1 (3.6)	0.6 (5.7)	2.3 (5.2)
end hypoxia	*2.4 (3.7)*	*1.6 (5.7)*	−19.7 (4.2)	−20 (3.7)	−18.5 (4.7)
end resuscitation	*0.72 (3.9)*	*0.9 (5.3)*	−14.7 (4.5)	−13.9 (4.1)	−11.7 (5.7)
2h>	4.2 (4.5)	0.8 (6)	−3.5 (5.8)	−5.3 (3.9)	−2.5 (7.7)
5h>	1.2(4.9)	−0.6 (7.3)	−2.7 (5.2)	−4.7 (4.5)	−0.1 (5.8)
9h>	2.1(6.1)	−4.2 (7.2)	−6.1 (4.8)	−6.3 (5.2)	−2.8 (7)
**MABP** mmHg start	43.3(3.4)	42.6 (17.3)	49.0 (8.4)	47.5 (7.8)	51.4 (8.3)
end hypoxia	*42.3 (2.9)*	*44.1 (4.8)*	22.5 (15)	22.8 (10.8)	22.0 (15.5)
endResuscitation	*43.2 (2.7)*	*45.6 (9.6)*	40.0 (15.8)	41.6 (12.1)	40.0 (11.7)
2h>	42.6(5.1)	44.8 (8.5)	41.8 (12.2)	36.8 (11.7)	40.5 (10.3)
5h>	45.7(16.4)	39.4 (9)	42.8 (10)	38.4 (10)	44.0 (9)
9h>	38.1 (7.1)	40.1 (14.7)	35.2 (6.7)	39.0 (10)	39.4 (14.7)
Heart rate start	137 (27)	149 (34)	153 (31)	165 (34)	151 (30)
end hypoxia	*136 (34)*	*150 (32)*	154 (34)	143 (35)	165 (37)
endResuscitation	*138 (32)*	*140 (28)*	177 (31)	192 (36)	170 (33)
pO_2_ kPa start	10.6 (1.4)	12.7 (1.9)	11.6 (1.6)	12.0 (1.3)	11.8 (2.0)
end hypoxia	*11.3 (1.2)*	*12.8 (1.3)*	5.3 (1.4)	5.0 (0.8)	5.1 (0.7)
endResuscitation	*11.5 (1.2)*	*61.4 (8.3)*	12.4 (2.0)	25.8 (2.7)	52.2 (15.4)
pCO2 kPa start	5.3 (0.8)	4.9 (0.7)	5.3 (0.4)	5.1 (0.6)	5.2 (0.8)
end hypoxia	*5.0 (0.6)*	*4.9 (0.8)*	8.9 (1.6)	8.8 (2.1)	8.6 (1.7)
endResuscitation	*5.0 (0.6)*	*4.7 (0.7)*	5.0 (0.7)	5.2 (0.8)	4.8 (1.3)

*Characterization of the study cohort before, directly after asphyxia and after reoxygenation. Values are presented as mean (±SD). Italic values show the control- and hyperoxia group at corresponding time points.*

There were significant dose-dependent differences in pO_2_ between the groups after oxygen supplementation: 100% vs. 40%, p<0.001; 40% vs. 21%, p = 0.005, and the hyperoxia group vs. 100%, 40% and 21%, p<0.001 (data presented in Tab.1).

Three piglets died after hypoxia, one in each group.

### Histopathology in the striatum, hippocampus, cortex and cerebellum

Grading of damage was assessed on the HE stained sections and divided into eight different categories as shown in Table 2. The mean scores are given in Table 3. One-way analysis of variance with Tukey's post hoc test revealed no significant differences at 9 h in the brain region-specific scores or total scores between the three hypoxia-reoxygenated groups or between them and the control group (Tab. 2 and 3). The degree of hypoxia (PaO_2_ at the end of hypoxia) correlated significantly with the histopathological score (r = 0.4, p = 0.002, n = 48). For the three hypoxia-reoxygenated groups (n = 32), there was no correlation between length of hypoxia and histopathology score (r = 0.1, p = 0.2–0.9). The evolution or degree of cell death 9 ½ h after the hypoxic event was more pronounced in the cortex, striatum and cerebellum than in the hippocampus.

**Table 2:** Grading of damage in striatum, hippocampus, cortex and cerebellum (HE stained sections).

Grade	Degree of damage
0	No necrosis
1/+	≤10% of tissue necrotic
2/+ (+)	∼20% of tissue necrotic
3/++	∼30% of tissue necrotic
4/++(+)	∼45% of tissue necrotic
5/+++	∼60% of tissue necrotic
6/+++(+)	∼75% of tissue necrotic
7/++++	90–100% of tissue necrotic

**Table 3:** Brain histopathology score.

	Control	21%	40%	100%	Hyperoxia
Cortex	1.42 (0.9)	1.45 (1.4)	1.71 (1.4)	1.70 (0.9)	0.36 (0.5)
Striatum	0.67 (0.8)	1.55 (1.7)	1.96 (1.5)	1.89 (1.3)	0.23 (0.4)
Hippocampus	0 (0)	1.0 (1.3)	1.17 (1.6)	0.7 (1.1)	0 (0)
Cerebellum	1.33 (1.2)	1.8 (0.9)	2.29 (0.8)	1.8 (0.9)	1.32 (0.8)

Mean scoring values (±SD) for the HE stainings.

n = 6, 10, 12, 10, 11 for the control, 21%, 40%, 100% and Hyperoxia groups.

There were no statistical difference between hypoxia-reoxygenated groups and the control group (p = 0.25–1.0) and between the control group and the hyperoxia group (p = 0.34 (cortex), p = 0.95–1.0 (striatum, hippocampus and cerebellum)).

One piglet in the hyperoxia group had severe brain edema and was difficult to evaluate by HE staining.

**Figure 1:**
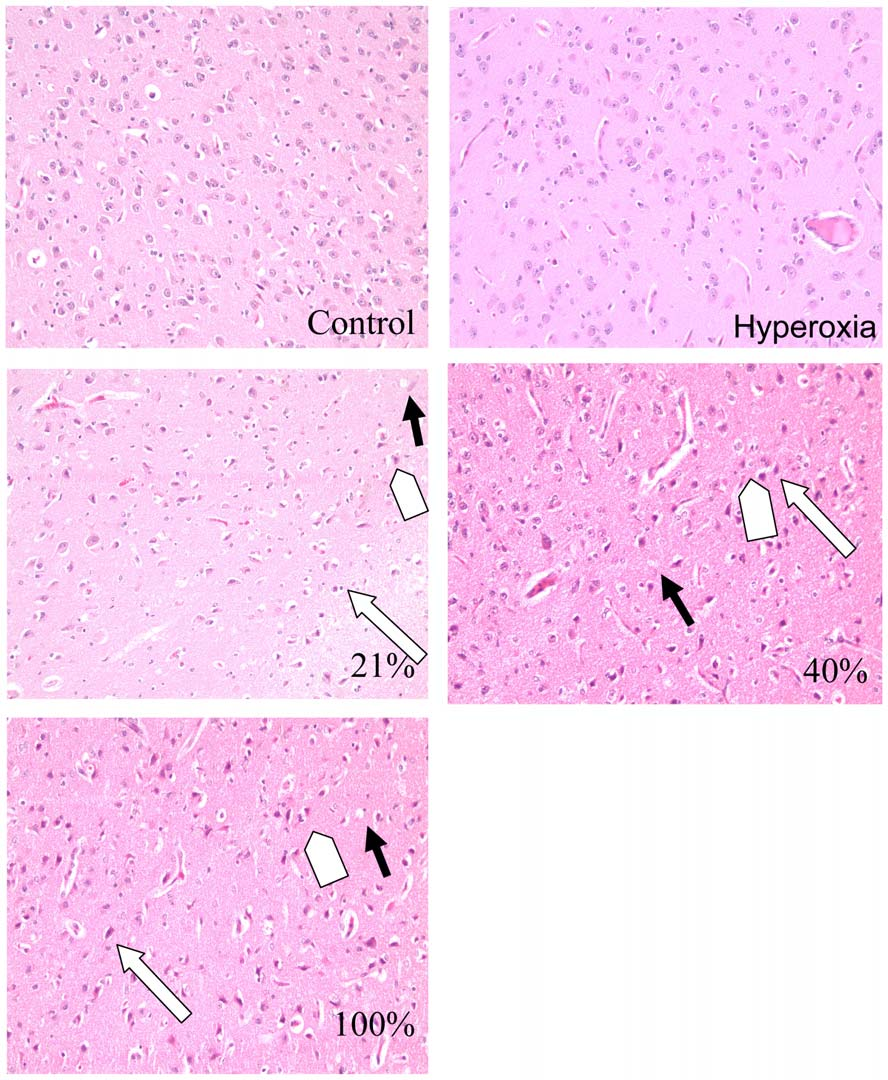
Brain histopathology. Typical morphological changes after hypoxia and reoxygenation. HE-stained sections from the corpus striatum with vacuolated neuropil (black arrow), shrunken neurons with pyknotic nuclei (white arrow), and eosinophilic neurons (arrow head) from one representative animal in each group together with HE stained sections from the control- and hyperoxia group. Obj. ×20.

Figure 1 contains a representative hematoxylin and eosin (HE) staining from each group.

### Metalloproteinase expression and activity

**Figure 2:**
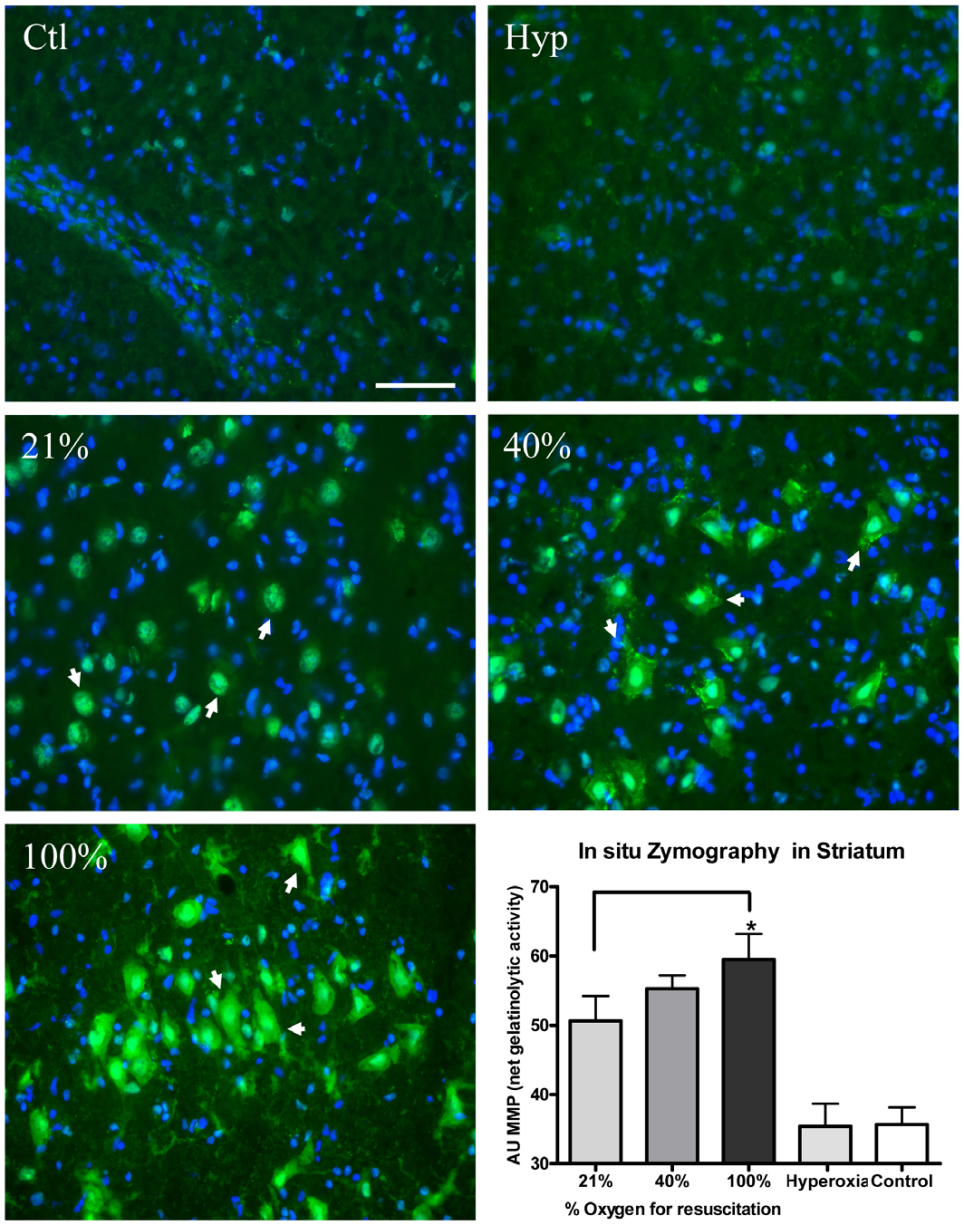
*In situ z*ymography in the corpus striatum. Net gelatinolytic activity increases in the striatum after hypoxia-resuscitation. Fluorescence photomicrographs of striatum sections showing *in situ* zymography in sham operated (Ctl) and hyperoxia (Hyp) controls and after reoxygenation with 21%, 40% or 100% O_2_. Overall, fluorescence signal representing proteolytic activity (green) increases after hypoxia-resuscitation in the entire tissue, but the most prominent changes occur in discrete neuronal populations (arrows) in a dose-response manner. Hoechst stain was used as a nuclear marker (blue). Scale bar: 150 µm. The graph represents the quantification of net gelatinolytic activity (in arbitrary units (AU) of fluorescence) for 21% (n = 8) 50.64 (10.1), 40% (n = 8) 55.29 (5.4), 100% (n = 9) 59.51 (11.1), hyperoxia (n = 6) 35.42 (8.0) and controls (n = 6) 35.67 (6.1). There was a significant increase in net gelatinolytic activity in the corpus striatum in all groups exposed to hypoxia-reoxygenation vs the control group (p = 0.024, p = 0.002 and p<0.001 for the 21%, 40% and 100% group). The hyperoxia-group was similar to the control group. Using *post hoc* multiple comparisons between group means (Fisher LSD), we found a significant increase in the 100% oxygen group compared to the 21% oxygen group (p = 0.043). Values are expressed as a mean (±SD), *p<0.05, **p<0.01.

**Figure 3:**
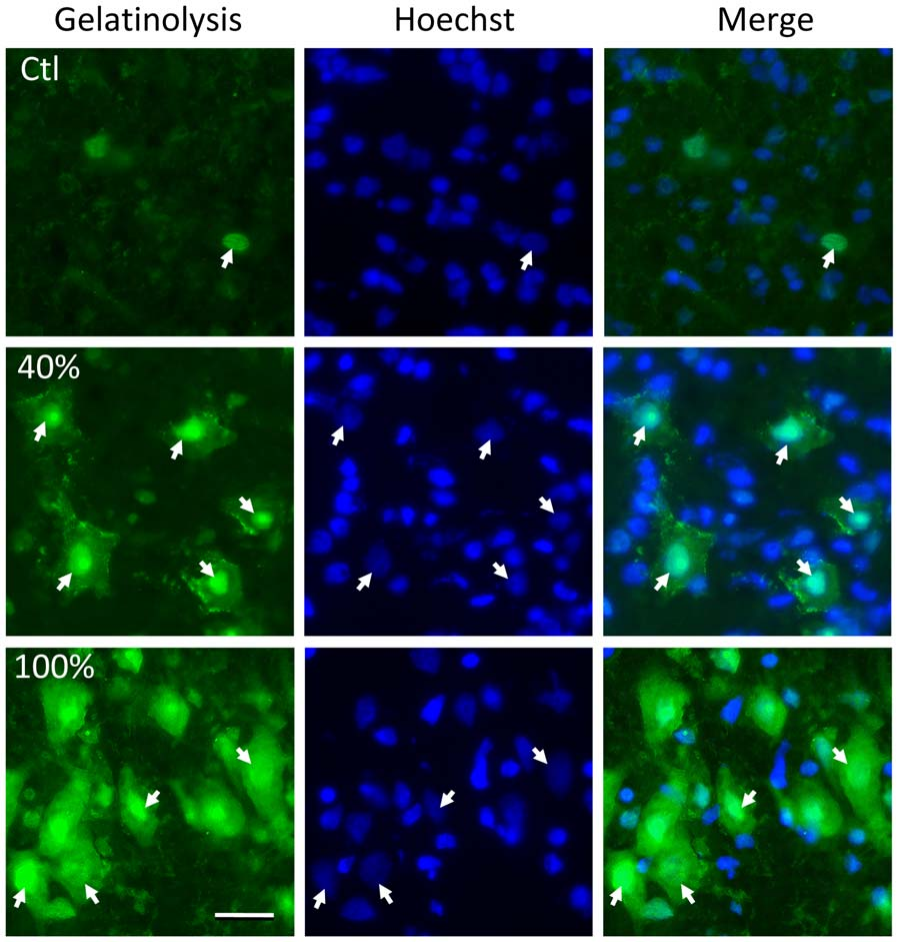
*In situ z*ymography. Net gelatinase activity at the nuclear level. High power magnification of fluorescence photomicrographs of the striatum sections showing *in situ* zymography (green) and nuclear marker Hoechst (blue) in sham operated (Ctl) and after reoxygenation with 40% or 100% O_2_. Note that, the number of cells showing intense gelatinolysis in the nucleus (arrows) augments with the concentration of oxygen. Scale bar 25 µm.

There was a significant increase in the net gelatinolytic activity in the corpus striatum in all groups exposed to hypoxia-reoxygenation vs the control group (p = 0.024, p = 0.002 and p<0.001 for the 21%, 40% and 100% groups), suggesting an up regulation of the MMP activity, particularly the gelatinases MMP-2 and/or MMP-9. Using post-hoc multiple comparisons between group means (Fisher LSD), we found a significant increase in the gelatinase activity of the 100% group compared with the 21% group (p = 0.043) (Fig. 2). A detailed observation of the tissue revealed that gelatinolytic activity in the 100% and 40% groups was markedly increased not only in the cytoplasm and extracellular space, but also in the nuclear compartment (Fig. 3), indicating that the increase in proteolytic activity took place throughout the entire neuron. Values are presented in the figure legend (Fig. 2).

**Figure 4:**
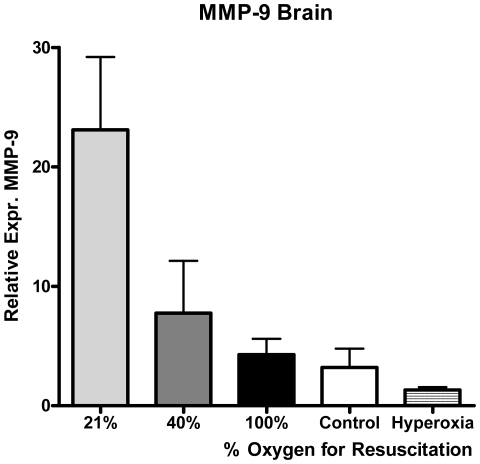
MMP-9 mRNA expression in the cortex. Relative mRNA MMP-9 expression was significantly increased in the 21% oxygen group vs. all the others: 21% (n = 10) 23.1 (19.3), 40% (n = 12) 7.8 (15.1), 100% (n = 10) 4.3 (4.2), hyperoxia (n = 11)1.8 (1.6), control (n = 6) 3.9 (4) with p = 0.029, 0.007, 0.001 and 0.017, respectively.

The relative mRNA expression of MMP-9 was significantly increased in the 21% group compared with all of the others (Fig. 4).

For MMP-2 mRNA, there was no difference between groups (data not shown).

### Changes in BDNF and caspase-3 after HI

**Figure 5:**
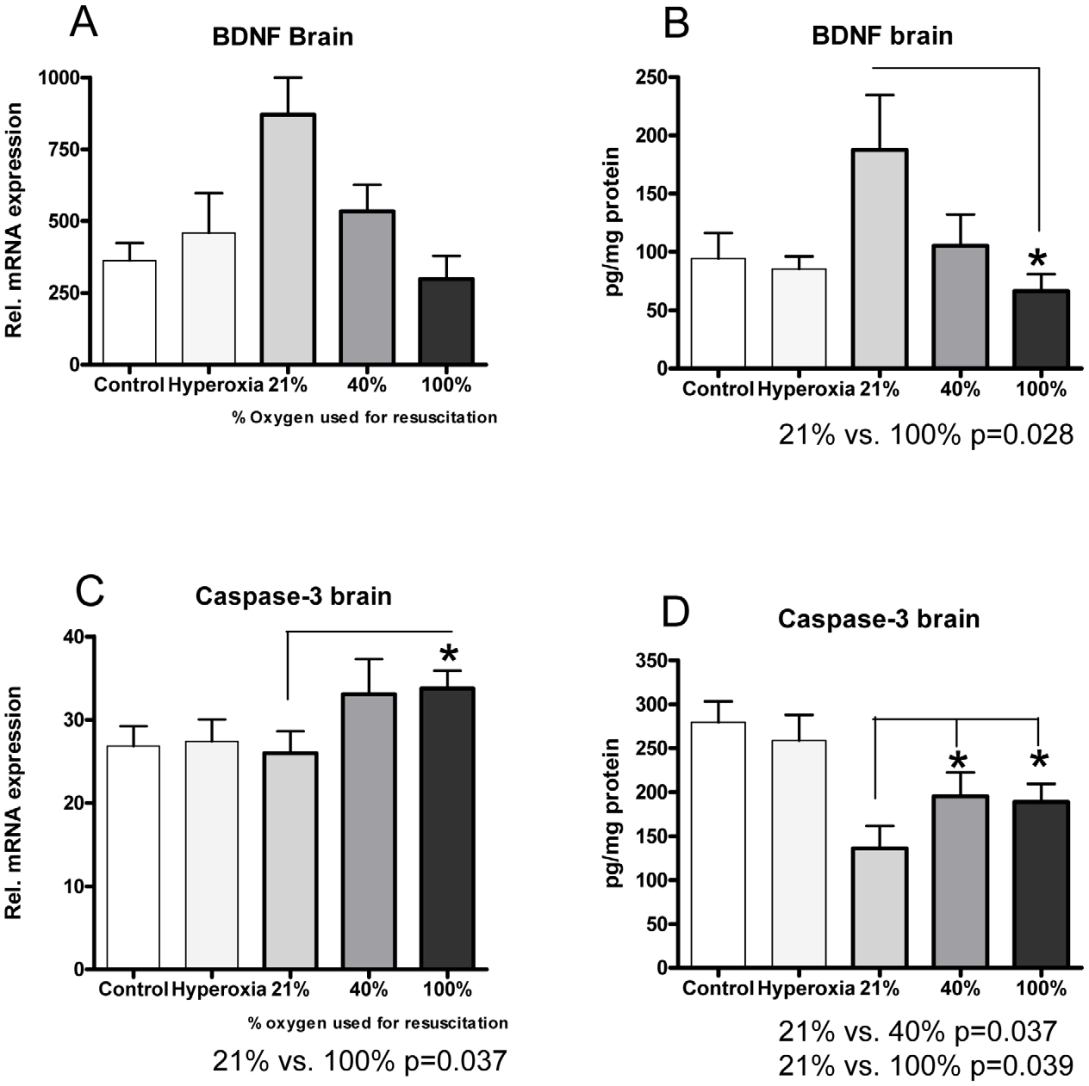
BDNF and caspase-3. **A:** For BDNF, the relative mRNA expression for each group was: 21% (n = 10) 914.6 (909), 40% (n = 10) 585.1 (642.8), 100% (n = 10) 466.5 (412.3) p = 0.15 for 21% vs. 100% due to great inter-animal variability.The hyperoxia group (n = 10) was equal to the control group (n = 6); 391.7 (374) vs. 406.2 (320). **B:** Immunohistochemistry (ELISA). BDNF (pg/mg protein) was 21% (n = 10) 187.5 (149.1), 40% (n = 12) 105.2 (93.3), 100% (n = 10) 66.4 (45.2). * p = 0.028 for 21% vs. 100%. The hyperoxia group (n = 11) was equal to the control group (n = 6); 85.3 (36.0) vs. 94.4 (53.5). **C:** For caspase-3, the relative mRNA expression for each group was: 21% (n = 10) (26.0 (8.4), 40% (n = 12) 34.0 (14.5), 100% (n = 9) 33.7 (6.5). *p = 0.037 for 21% vs. 100%. The hyperoxia group (n = 11) was equal to the control group (n = 6); 27.4 (8.7) vs. 31.2 (11.7). **D:** Immunohistochemistry (ELISA). Caspase-3 (pg/mg protein) was 21% (n = 10)136.1 (80.6), 40% (n = 12)195.1 (94.6), 100% (n = 10)188.9 (64.8). * p = 0.037 for 21% vs. 40% and *p = 0.039 for 21% vs. 100%. The hyperoxia group (n = 11) was equal to the control group (n = 6); 258.7 (97.5) vs. 279.6 (58.0). Caspase-3 expression levels correlated negatively with BDNF expression levels (r = −0.49, p = 0.024). Values are expressed as mean (±SD), *p<0.05.

There was a clear inverse correlation between BDNF and caspase-3 in the three hypoxia-reoxygenated groups, (r = −0.49, p = 0.024) (Fig. 5 A–D).

There was a 2.5 fold increase in BDNF expression and a 2 fold increase in BDNF activity in the 21% group vs. the control group. In the groups exposed to HI, there was an increase in expression of caspase-3 when supplementary oxygen (40% or 100%) was used for resuscitation. Caspase-3 activity fell after HI, but the groups resuscitated with 40% and 100% oxygen had a smaller fall compared with the 21% group (and thereby increased activity levels).

Exposure to 30 min of hyperoxia without preceding hypoxia revealed values similar to the control group for both relative gene expression and activity (pg/mg protein).

The study revealed no gender differences.

## Discussion

Histopathology revealed no significant increase in early brain damage in the HI groups.

This study demonstrated that net gelatinolytic activity in the corpus striatum from piglets resuscitated with supplementary oxygen was increased compared with controls, and the increase was significantly higher in the 100% group than in the 21% group. In keeping with these data, caspase-3 expression and activity was 30–40% higher in the 100% group than in the 21% group, while BDNF activity was decreased by 65%, suggesting an overall homeostatic imbalance that might hint a poor neurological outcome.

### Histopathology

Nine and a half hours after the hypoxic event and nine hours after resuscitation, areas with vacuolated neuropil, shrunken neurons with pyknotic nuclei and scattered eosinophilic neurons were representative of early neuronal death. We could not uncover histopathological differences related to the percentage of oxygen used during resuscitation. In contrast, recent work [28] demonstrates that 100% oxygen resuscitation increased HI lesion volumes compared with 21% oxygen in a neonatal rat model measured with T2 weighted MRI 24 h after resuscitation. The apparent discrepancy between these studies may stem from the use of different models; indeed, the piglet model appears as more physiological and closer to the human case than the rodent model, which may account for a different spatio-temporal evolution of damage. In this context, the 9½ h follow-up time after the HI injury may be too short a time to reveal clear changes at the histopathological level in our model, and consequently it is difficult to indubitably predict the effect of HI at later time points. Usually, after HI and resuscitation cell death continue to progress for days and even weeks [29] and neurochemical changes may persist for several days [30]. The long-term neurological deficit must be assessed to determine the efficacy of therapeutic interventions in asphyxiated neonates and ideally, it should be evaluated together with short-term mortality and neurofunctional deficit [31].

Other investigators have been evaluated neuronal injury at 48 to 72h after HI [32]–[36]. However, these models differed from our present one regarding anesthetics, the hypoxic event, and the grade of achieved hypotonia and subsequent impaired cerebral circulation. These factors may contribute to increased mortality with long-term survival seen in the current model. We are working on modifications to get higher survival rates at later time points in order to perform studies of sufficient duration to better ascertain the final degree of injury.

### Metalloproteinase expression and activity

Resuscitation after global HI resulted in increased net MMP gelatinase activity compared with controls, as well as a stepwise increase according to the percentage of oxygen given for resuscitation. The primarily neuronal localization of the net MMP gelatinase activity after global HI and resuscitation is in agreement with previous studies at relatively early time points after kainate-induced seizures and global cerebral ischemia [13], [37]. The increase in net gelatinolytic activity related to the fraction of inspired oxygen (FiO_2_) used for resuscitation is in accordance with a study by Munkeby and collaborators [15] that revealed strong up regulation of the MMP gelatinolytic activity as early as 2½ h after hypoxia-resuscitation. Taken together, these data suggest that increases in net gelatinase activity are related to later neuronal demise. In this context, early cytotoxic up regulation of MMP-9 levels have been associated with neuronal death in the ischemic and epileptic rodent brain [12], [13], and early MMP-9 up regulation in reactive microglia after ischemic episodes has been suggested to underlie the neurotoxic/inflammatory effect of the enzyme assessed in adult [37] and neonatal rat models [10]. The effect of MMP inhibitors further links early MMP activity to later cell death. Chen et al. [38] recently found that inhibition of MMPs provides neuroprotection against HI in a neonatal rat model; administrating a broad-spectrum MMP inhibitor reduced brain atrophy 2 weeks following neonatal HI and also improved neurological function at 7 weeks post-HI.

Measuring net gelatinase activity at 9½ h after hypoxia as we did in this study is a good time point to measure, considering that Ranasinghe and collaborators [14] recently showed that gelatinase activity was increased in the brain within 6 h after HI in a newborn rat model. Most interestingly, our study revealed an increase when 100% oxygen was used for resuscitation, emphasizing the potential harmful role of oxygen in newborn resuscitation. Furthermore, at high oxygen concentrations (40% and 100%), we observed conspicuous gelatinase activity at the nuclear level. These findings are in keeping with recent data demonstrating the presence of various MMPs in the nucleus of neural cells [39], [40], and a particular correlation between nuclear MMP-9 and neuronal apoptosis and DNA fragmentation in an ischemic brain injury model [41].

Thus, our observation of distinct net gelatinase activity at the nuclear level could interfere with oxidative DNA repair by cleaving DNA repair enzymes [41].

The increase in net gelatinolytic activity found in the striatum of groups receiving supplementary oxygen for resuscitation stands in contrast to the decrease of MMP-9 and preservation of MMP-2 mRNA levels found in these experimental groups. We found similar results in the livers of the same animals, showing a linear increase in gelatinolytic activity proportional to oxygen supply that was not accompanied by significant changes in MMP levels [27]. In contrast, previous data from our laboratory demonstrated the up regulation of MMP-9 and/or MMP-2 levels in the brain [15] and lungs [42] as early as 2.5 h post-resuscitation, with a good correlation between MMP levels and *in situ* zymography activity in the lungs. Furthermore, the upregulation of MMP-2 expression in the basal ganglia of piglets 6 h after hypoxia-resuscitation has also been reported [16], but there were no significant differences between the reoxygenated groups. The apparent discrepancy between the mRNA and *in situ* zymography data presented here suggests that changes in net proteolytic activity may occur without changes in MMP mRNA or even protein levels at 9 h after resuscitation. Net proteolytic activity results from multistep regulatory processes, including the proteolytic balance between MMPs, tissue inhibitors of MMPs (TIMPs) present in the tissue or the post-translational regulation of MMP activation. Accordingly, previous work has demonstrated that oxidative nitrosylation concomitant to cerebral ischemia activates at least MMP-9 and leads to neuronal death [12]. Thus, it is conceivable that the decrease/stabilization of the MMP-9/MMP-2 mRNA levels in the brain and liver at 9 h post-resuscitation represents a delayed homeostatic response of the organism challenged by an early up regulation of MMP levels and a sustained increase in net proteolytic activity in a highly oxidative environment. Pure oxygen could also have an effect on the expression and activity of TIMPs and other factors that modulate the final proteolytic outcome with uncertain overall effect on the brain. MMPs may clearly act as pleiotropic factors that convey both beneficial and detrimental effects in the nervous system [43]. Early after an injury they contribute for instance to the opening of the blood-brain barrier and initiation of cell death by apoptosis, whereas, during the second stage of injury, they are involved in angiogenesis and neurogenesis [44] and promote plasticity and recovery [17].

### The rise in BDNF after HI is attenuated if supplementary oxygen is used for resuscitation

This is in accordance with a study in rats showing that hyperoxia reduces mRNA levels for BDNF and three other neurotrophins [9]. BDNF plays a critical role in brain development, neuroplasticity, learning and memory [18], [20], [45]. Among the neurotrophins, BDNF has shown independent and markedly neuroprotective effects against neonatal HI injury *in vivo* [46] and BDNF can protect neurons against oxidative damage [47].

Given the negative correlation between BDNF and caspase-3 activity and that the neurotrophin has been shown to almost abolish hypoxia-ischemia induced caspase-3 activation [19], it is possible that the increased BDNF/caspase-3 ratio in the 21% oxygen group accounts for a higher degree of neuroprotection as compared with the 40% and 100% groups. This hypothesis finds support in recent data demonstrating that intraventricular injection of BDNF to neonatal rats prior to HI results in less tissue loss in the hippocampus, cortex and striatum and also results in less spatial memory deficits than when given as a pretreatment vehicle [20]. The results of our study, showing the stepwise attenuation of the rise in BDNF after HI with increasing oxygen concentrations, raise concerns about a lower level of endogenous neuroprotection if supplementary oxygen is used for newborn resuscitation.

### Higher levels of Caspase-3 if supplementary oxygen is used for resuscitation

Our data show higher caspase-3 levels along with oxygen concentration due to a attenuated reduction in caspase-3 activity and higher mRNA expression levels compared with 21% oxygen. This is in agreement with previous studies [9], [48] in a newborn rat model. That study demonstrates that apoptosis of oligodendrocytes and neurons in response to hyperoxia correlates with a significant higher caspase-3 activity in these cells after 100% oxygen exposure when compared with 21% oxygen exposure. In contrast, Mendoza-Paredes and collaborators [49] found caspase-3 to be decreased after 100% oxygen was used to treat repeated intermittent apnea in newborn pigs. However, these pigs were older (2 to 4 days), were primed to hyperoxia during anesthesia-introduction, went through 10 episodes of intermittent hypoxia/hyperoxia and were followed for only 6 h.

In the immature brain there is a basal activity of caspase-3 and both hypoxia and reoxygenation can alter this [50].We found relatively high caspase-3 activity in the control- and hyperoxia- groups, probably associated with the role of this protease in developmental programmed cell death [50]. Caspase-3 protein and mRNA decrease by more than 80% as brain growth spurt levels out [24]. As previously mentioned, BDNF may almost abolish HI-induced caspase-3 activation *in vivo* [19]. Thus, the decrease in caspase-3 activity seen in our study after HI could in part be due to BDNF effects. Another explanation could be that global ischemia induces endogenous caspase inhibitors, such as IAP proteins (inhibitor of apoptosis), that can bind and inhibit activated caspases [51]. Additionally, severe hypoxia, secondary energy failure, impaired mitochondrial function and lack of ATP could eventually interrupt the apoptotic cascades to the extent that the level of caspase-3 activity is brought down to a level close to background [52].

The three HI groups were treated equally aside from the 30 min of graded FiO_2_ used for reoxygenation. We interpret the 30–40% higher values we found for caspase-3 expression and activity in the 100% group vs. the 21% group as unfavorable towards neuronal cell survival. Activated caspase-3 cleaves numerous intracellular proteins; it also cleaves and inactivates nuclear enzymes such as the DNA repair enzyme poly(ADP-ribose) polymerase (PARP), whose inactivation could lead to the cessation of cellular DNA repair [53], [54].

### Supplementary oxygen

Hyperoxia without preceding hypoxia did not bring about significant changes compared with the otherwise equally treated control group in this study. Because our follow-up time was just 9 h, the long-term effects of the 30 min of hyperoxia were not sufficiently explored. However, in a different study arm using the same model, we found that hyperoxia alone decreased the expression of VEGFR2 and TGFBR3 in liver tissue compared with the otherwise equally treated control group. These two genes are important for angiogenesis and tumorigenesis, and the fact that they were down-regulated 9 h after the 30 min of exposure to 100% oxygen may be of concern [27].

The rationale for choosing 40% was that some clinics have started to use intermediate oxygen concentrations for newborn resuscitation. Our group has previously shown a dose dependent increase in hydroxyl radical attack and an increase in DNA damage after 40% or 60% oxygen was used for resuscitation [55]. We therefore considered it interesting and clinically relevant to explore differences with a relatively minor increase in FiO_2_ from 21% to 40%.

### The piglet model as a model of choice to extrapolate to human neonates

The maturation of the piglet brain is similar to that of a term infant [26], and the brain growth-spurt period is also comparable, with a peak occurring around term. The brain growth-spurt is probably a period of enhanced vulnerability due to all the developmental events taking place; anatomical, metabolic and behavioral [56]. Newborn pigs' size permits the use of the same intensive care equipment used for newborn babies. This study has been done in a neonatal, not perinatal, model of hypoxia-reoxygenation. Therefore, some caution must be taken when interpreting the current findings in the context of birth asphyxia.

### Conclusions

The present data raise concerns about resuscitation with 100% oxygen after perinatal hypoxia-ischemia since this procedure may trigger an imbalance between neuroprotective and neurotoxic mechanisms when compared with 21% oxygen treatment. Although attenuated BDNF levels and overall increased activities of potentially neurotoxic caspase-3 and MMPs may promote neuronal damage, this was not unequivocally detected in the present study 9h after reperfusion. Future studies should therefore be of sufficient duration in order to ascertain if this early homeostatic imbalance compromises the neurodevelopmental plasticity and repair outcome.

## Materials and Methods

### Approval

The Norwegian Council for Animal Research approved the experimental protocol. The animals were cared for and handled in accordance with the European Guidelines for Use of Experimental Animals, by certified FELASA (Federation of European Laboratory Animals Science Association) researchers.

### Surgical preparation and anesthesia

Forty-nine newborn Noroc (LYxLD) pigs were included in the study. Inclusion criteria were 12–36 h of age, Hb>5g/dL and good general condition. The piglets were anaesthetized, orally intubated, ventilated and surgically prepared as described by Andresen et al. [57]. A continuous IV infusion of Salidex (saline 0.3% and glucose 3.5%, 10 mL·kg^−1^·h^−1^) was given until hypoxia. Fifteen minutes after the start of resuscitation, Salidex was reduced to 5mL·kg^−1^·h^−1^. After a suprapubic catheter was inserted at 5 h post-resuscitation, some adjustments were made to the infusion rate in response to urine production.

### Experimental protocol

After 60 min of stabilization, the piglets were randomly assigned to either undergo global hypoxia and resuscitation (Groups 1–3), to receive 100% oxygen for 30 min (Group 4) or to be in the control group (Group 0), going through the same procedures and observation times (anesthesia, surgery, ventilation and sample collection), but without hypoxia or hyperoxia.

For groups 1–3, hypoxemia was achieved by ventilation with a gas mixture of 8% O_2_ in N_2_ until either the base excess (BE) reached −20 mM or the mean arterial blood pressure decreased to 15 mm Hg (impaired brain circulation). CO_2_ was added during hypoxemia aimed at a PaCO_2_ of 8.0–9.5 kPa, to imitate perinatal asphyxia. Before the start of resuscitation, the hypoxic piglets were block-randomized into three different groups. Resuscitation was performed for 30 min with either 21% O_2_ (Group 1, n = 10), 40% O_2_ (Group 2, n = 12) or 100% O_2_ (Group 3, n = 10). At the corresponding time point Group 4 (n = 11) received 100% oxygen for 30 min. Thereafter, the piglets were observed for 9 h (receiving 21% O_2_ and normocapnia (PaCO_2_ 4.5–5.5 kPa)) with continuous surveillance of blood pressure, saturation, pulse, temperature and blood gas measurements. The control group (n = 6) received 21% oxygen throughout the experiment. All blood drawn for tests was replaced by saline at a volume of 1.5 times the volume drawn. A suprapubic catheter (BD Venflon Pro 20GA, 1.1mm×32 mm. Dickinson Infusion Therapy AB, Helsingborg, Sweden) was inserted under sterile conditions after 5 h, and urine was collected to evaluate urine production. At the end of the observation time, the animals were given an overdose of pentobarbital (150 mg/kg IV). The brain and cerebellum were immediately removed and sagitally divided, and the left half was placed in 4% buffered formalin. From the right half, specimens from the fronto-parietal cortex, the corpus striatum and the cerebellum were frozen in liquid nitrogen and stored at −70°C until subsequent analysis.

***In situ*** **zymography** was performed to localize net gelatinolytic activity in brain sections from the corpus striatum, with a few modifications to a method previously described for brain tissue [37]. *In situ* zymography is commonly used as an index of net metalloproteinase activity resulting from the balance between gelatinases (principally MMP-9 and MMP-2) and the TIMPs present in the sample. Sections of fresh frozen brain tissue (20µm thick) from the corpus striatum were generated using a cryostat (Leica CM3050S, Nussloch, Germany). Nonfixed brain sections were incubated for 2 h at 37°C in a humid dark chamber in a reaction buffer that contained 0.5 M Tris-HCl, 1.5 M NaCl, 50 mM CaCl_2_, 2 mM sodium acide (pH 7.6) and 80 µg/mL FITC-labeled DQ-gelatin (EnzCheck collagenase kit; Molecular Probes, Eugene, OR) that was intramolecularly quenched. After the incubation, the tissue was fixed in 4% paraformaldehyde (Acros, Elancourt, France), incubated for 5 min with 0.5 µg/mL Hoechst 33258 (Molecular Probes, Leiden, the Netherlands) and mounted in fluorescent mounting medium (Dako, Carpinteria, CA). Some sections were incubated with 1 mM phenanthroline (Molecular Probes), a broad-spectrum metalloproteinase inhibitor. Samples were observed with a fluorescent microscope (E800; Nikon, Champigny-sur-Marne, France) equipped with FITC and DAPI filters, and images were analyzed using an ORCA camera (Hamamatsu) and the Lucia software (Nikon). Gelatin-FITC cleavage by tissue gelatinases releases quenched fluorescence representative of net proteolytic activity. Sections incubated without DQ-gelatin were not fluorescent. We used 6 to 9 piglets per experimental group and analyzed three slices per animal.

### Pathological examination

Tissue blocks (0.5 cm thick) from the cortex, striatum, hippocampus and cerebellum were embedded in paraffin, sliced in 4 µm thick sections and stained with hematoxylin and eosin (HE). In the cerebrum hypoxic/ischemic damage was defined as areas with vacuolated neuropil and dark, shrunken or eosinophilic neurons with pyknotic nuclei; in the cerebellum eosinophilic Purkinje cells were the indicators of hypoxic/ischemic damage. The severity of damage was assessed on the HE stained sections and graded with 0.5-intervals from 0.0–4.0 giving an eight step scale as presented in Table 2.

### Tissue preparation for real-time PCR

Twenty milligrams of tissue, which had been kept in RNA safer, was placed in MagNA Lyser Green bead tubes (Roche Diagnostics GmbH, Germany) and TRK lysis buffer. Total RNA from the supernatant was prepared using a Total RNA Kit from E.Z.N-A., and was treated with DNase I (E.Z.N-A., Omega Bio-tek, USA). Extracted total RNA was quantified using a ND-1000 spectrophotometer (NanoDrop Technologies, Inc., USA). Total RNA (2–3 µg) was reverse transcribed into cDNA employing the High Capacity cDNA Archive Kit (Applied Biosystems Inc.) in a PTC-100 thermal cycler (MJ Research, USA) according to the manufacturer's protocol. Real-time PCR was performed with 20 ng cDNA for target genes and a housekeeping gene (PPIA), employing the SYBR Green PCR Master mix in an ABI PRISM® 7300 Sequence Detection System using universal instrument settings. The following primer concentrations were used:

MMP-2: (GenBank accession no. NM_214192) (forward primer: 5′- GGCTTGTCACGTGGTGTCACT -3′; reverse primer: 5′-ATCCGCGGCGAGATCTTCT-3′), MMP-9: (GenBank accession no. NM_001038004) (forward primer 5′-GAAGCTTTAGAGCCGGTTCCA-3′; reverse primer 5′-GGCAGCTGGCAGAGGAATATC-3′), BDNF: (GenBank accession no. NM_214259.1) (forward primer: 5′- AGC GTG TGC GAC AGC ATT AG-3′; reverse primer 5′-GTC CAC TGC CGT CTT TTT ATC C-3′), caspase-3:(GenBank accession no. NM_214131) (forward primer 5′- GACGGACAGTGGGACTGAAGA-3′; reverse primer 5′-GCCAGGAATAGTAACCAGGTGC-3′) and the housekeeping gene PPIA: (GenBank accession no. MN_214353) (forward primer: 5′-ATACGGGTCCTGGCATCTTG-3′; reversed primer: 5′AACTGGGAACCGTTTGTGTTG-3′).

Brain tissue from the fronto-parietal cortex was used because previous work detected caspase-3 activation as early as 6h after hypoxia in the cortex and at 12–18 h in the hippocampus of P7 rats [19].

Relative expression was determined by the comparative C_T_ method of relative quantification (RQ) and calculated with the arithmetic formula 2^−ΔCt^, where ΔCt is the normalized signal level in a sample. (ΔCt = Ct of target gene−Ct of endogenous control gene) [58].

Real-time quantitative RT-PCR was performed on samples from Group 0 (controls), n = 6; Group 1, n = 10; Group 2, n = 12; Group 3 n = 10; and Group 4, n = 11.

Values are expressed as mean (±SD).

### Immunoassays

Fifty milligrams of prefrontal cortex was homogenized and proteins were extracted using ice-cold lysis buffer (Tris-HC (pH 7.5) containing 1% NP-40 and a protease inhibitor cocktail (without EDTA) and MagNA Lyser Green Beads (Roche Diagnostics GmbH, Mannheim Germany). Samples were then homogenized for 50 sec at 6500 rpm, incubated on ice for 15 min at 4°C, and then subjected to sonication for 1 min before finally being centrifuged at 12000×G for 15 min at 4°C. The supernatants were retained, spun for five min and the protein concentration of the samples was measured using the BCA method (Pierce, Cheshire, UK).

Quantikine® immunoassays were used to detect BDNF (DBD00) and caspase-3 (KM300). Quantikine® KM 300 measured activated caspase-3 protein. We tested human kits first and found them to be acceptable for porcine samples. The results were adjusted to the protein-content in the samples ( = pg BDNF or caspase per mg of protein in the samples). Values are expressed as the mean (±SD).

### Statistics

Statistical calculations were performed using the SPSS 15.0 statistical package for Windows (Chicago, IL). Values are expressed as the mean ± SD. One-way analysis of variance with Tukey's post-hoc test was used to examine differences between groups. For *in situ* zymography in the corpus striatum, one-way ANOVA with an LSD post-hoc test was used to examine differences between group means. The relationship between variables was studied using Pearson's product-moment correlation coefficient.

Statistical difference was accepted at p<0.05.
